# Emerging role of exosomal microRNA in liver cancer in the era of precision medicine; potential and challenges

**DOI:** 10.3389/fmolb.2024.1381789

**Published:** 2024-06-27

**Authors:** Tarek El Hayek, Osama Abdulwahab Alnaser-Almusa, Sulaiman Mamoun Alsalameh, Maya Taofik Alhalabi, Ahmad Nedal Sabbah, Eman Abdullah Alshehri, Tanveer Ahmad Mir, Naresh Kumar Mani, Khaled Al-Kattan, Raja Chinnappan, Ahmed Yaqinuddin

**Affiliations:** ^1^ College of Medicine, Alfaisal University, Riyadh, Saudi Arabia; ^2^ Tissue/Organ Bioengineering and BioMEMS Laboratory, Organ Transplant Centre of Excellence (TR&I-Dpt), King Faisal Specialist Hospital and Research Centre, Riyadh, Saudi Arabia; ^3^ Centre for Microfluidics, Biomarkers, Photoceutics and Sensors (μBioPS), Department of Biotechnology, Manipal Institute of Technology, Manipal Academy of Higher Education, Manipal, India; ^4^ Lung Health Center Department, Organ Transplant Centre of Excellence, King Faisal Specialist Hospital and Research Centre, Riyadh, Saudi Arabia

**Keywords:** exosomes, exosomal miRNAs, hepatocellular carcinoma(HCC), non-coding RNAs, cancer biomarker, liver cancer

## Abstract

Exosomal microRNAs (miRNAs) have great potential in the fight against hepatocellular carcinoma (HCC), the fourth most common cause of cancer-related death worldwide. In this study, we explored the various applications of these small molecules while analyzing their complex roles in tumor development, metastasis, and changes in the tumor microenvironment. We also discussed the complex interactions that exist between exosomal miRNAs and other non-coding RNAs such as circular RNAs, and show how these interactions coordinate important biochemical pathways that propel the development of HCC. The possibility of targeting exosomal miRNAs for therapeutic intervention is paramount, even beyond their mechanistic significance. We also highlighted their growing potential as cutting-edge biomarkers that could lead to tailored treatment plans by enabling early identification, precise prognosis, and real-time treatment response monitoring. This thorough analysis revealed an intricate network of exosomal miRNAs lead to HCC progression. Finally, strategies for purification and isolation of exosomes and advanced biosensing techniques for detection of exosomal miRNAs are also discussed. Overall, this comprehensive review sheds light on the complex web of exosomal miRNAs in HCC, offering valuable insights for future advancements in diagnosis, prognosis, and ultimately, improved outcomes for patients battling this deadly disease.

## 1 Introduction

Hepatocellular carcinoma (HCC) is the most common type of primary malignancy of the liver, occurring most frequently in patients with underlying chronic heaptic dysfunctions. HCC is the fourth most prevalent cause of cancer-related deaths worldwide and the sixth most common malignant tumor worldwide (Craig et al., 2020). The healthcare and economic burden of HCC continues to rise, and its incidence is projected to exceed over one million cases by 2025 (Estes et al., 2018). HCC is a severe threat to people’s physical and mental health because of its covert beginnings, high degree of malignancy, and poor prognosis. It is also considered a primary cause of mortality for individuals with liver cirrhosis and encephalopathy (Aravalli et al., 2008; Pinzani et al., 2011). Chronic infection with the hepatitis B virus (HBV), hepatitis C virus (HCV), alcoholic liver disease, and nonalcoholic steatohepatitis (NASH)/nonalcoholic fatty liver disease (NAFLD) are the most frequent etiologies causing liver cirrhosis, which in turn predispose to HCC transformation (Daher et al., 2023). Other less common risk factors responsible for triggering HCC include Wilson’s disease, autoimmune hepatitis, porphyria, one antitrypsin deficiency, steroid hormone abnormalities, familial hemochromatosis, and dietary aflatoxins (Granata et al., 2006; Miceli et al., 2009; El-Serag, 2011; Sukocheva, 2018) The overall initiation, development and progression of HCC is a multi-step and intricate process involving hepatocyte regeneration and necrosis linked to fiber deposition, as well as ongoing inflammatory damage. The high molecular heterogeneity of HCC is explained in detail by the combination of epigenetic modification and somatic genome mutation accumulation (Schulze et al., 2016). Therefore, the search for effective biomarkers for the detection and diagnosis of HCC has great clinical utility (Liu X.-N. et al., 2019). The detection of multiple hepatocellular carcinoma stem cell surface biomarkers (CD44, CD90, CD133/2 and OV-6) using electrochemical immunosensors has been demonstrated (Eissa et al., 2017). HCC can be treated primarily with surgery, transplantation, ablation, transarterial chemoembolization (Llovet et al., 2002; Lo et al., 2002; Llovet and Bruix, 2003), and drug therapy with agents such as Regorafenib and Lenvatinib (Bruix et al., 2017; Kudo et al., 2018).

**SCHEME 1:**
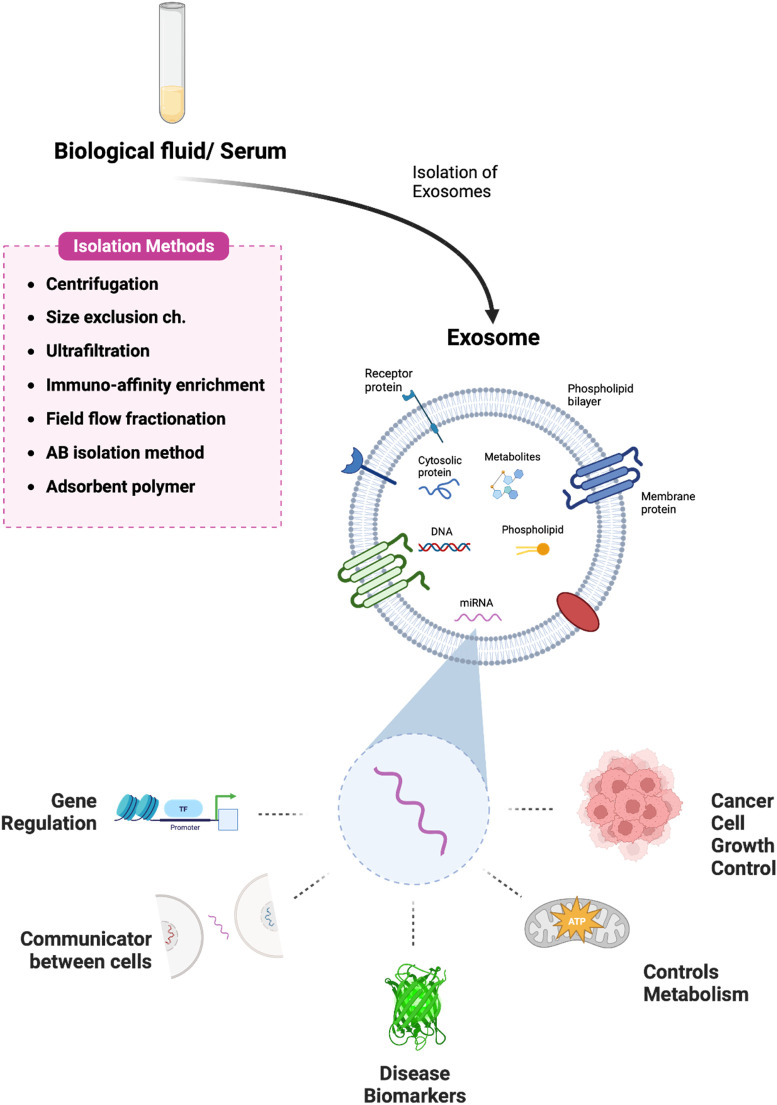
Pictorial representation of exosome isolation and the various roles of exosomal-miRNA. Exosomes are isolated from biological fluids or serum samples using various methods and the multiple functions of microRNA derived from exosomes have been illustrated. Created with BioRender.com.

**FIGURE 1:**
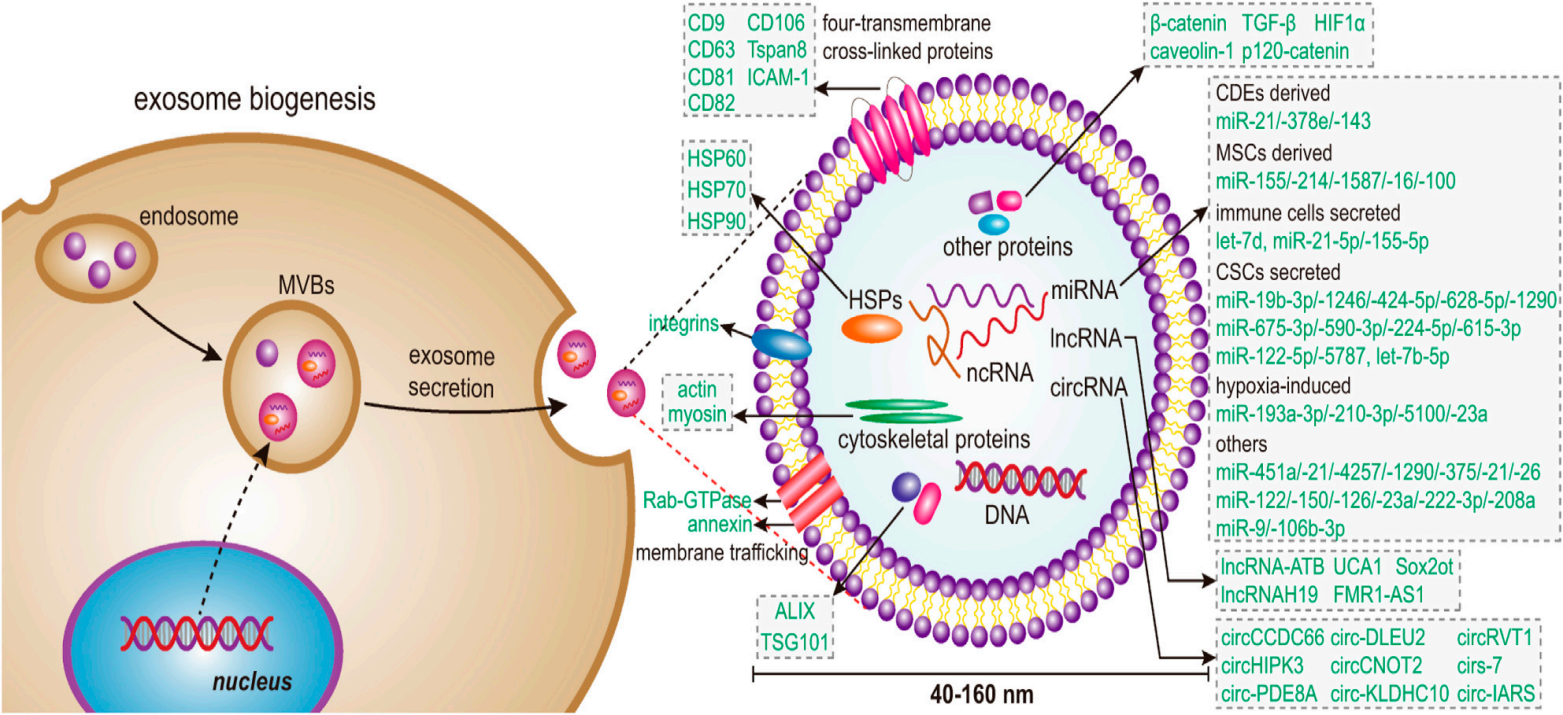
Schematic illustration shows that exosome biogenesis in cells begins with budding to the inner side of the plasma membrane, which allows for the generation of early endosomes. Next, the endosome sorting complex protein family required for transport (ESCRT) promotes the formation of late endosomes that collect various microparticles and apoptotic contents, including nucleic acids, proteins, and lipids, ultimately leading to the formation of multivesicular bodies (MVBs). Finally, MVBs amalgamate with the plasma membrane to form exosomes, which are secreted into the extracellular environment by exocytosis. Adapted from Dai et al., 2020 with copyright permission under the terms of the CC-BY-NC-ND 4.0 license.

Exosomes are 50–150 nm-diameter nanovesicles that are released into the extracellular milieu by fusing with the cell membrane (Théry et al., 2002). Tumor cells can influence nearby cells by means of exosomes, creating an environment that is conducive to tumor growth (Wu et al., 2019). Meanwhile, immune cells and matrix cells (like stellate cells and mesenchymal stem cells) can act against tumor cells by exosomes to encourage or prevent carcinogenesis (Zhou et al., 2017). Methods for isolating exosomes and the main roles of exosomal microRNAs are shown in Scheme 1. Exosomes contain a variety of genetic material such as mRNA, microRNAs, and other noncoding RNAs as well as proteins. (Figure 1) (Dai et al., 2020). They have crucial significance in chemical resistance, angiogenesis (Cho et al., 2012) epithelial-mesenchymal transition (EMT) (Tauro et al., 2013), and tumor metastasis (Kahlert and Kalluri, 2013) because they mediate signal pathways in recipient cells and are involved in intercellular communication and microenvironment regulation.

MicroRNAs are a class of non-coding RNAs that range in length from 17 to 24 nt. They participate in post-transcriptional control by causing the RNA-induced silencing complex (RISC) to degrade target mRNA or stop its translation by forming complementary base pairings with it (Ruvkun, 2006; Chen S. et al., 2017). Numerous biological activities, such as cell division, proliferation, migration, and the start and progression of disease, have been linked to microRNA (Gee et al., 2008; Tay et al., 2008; Kota et al., 2009; Png et al., 2012). Exosomal microRNA expression imbalances can hasten the course of a disease and impact the pathophysiological state of tumors. Additionally, the occurrence and development of tumors are closely linked to the aberrant expression of these microRNAs (Kosaka et al., 2013; Sun et al., 2018). According to the most latest studies, exosome-mediated microRNAs play a crucial role in the onset and progression of liver cancer (Li and Xu, 2019). As a result, the identification of serum exosomal microRNA for early diagnosis and prognostication of HCC becomes appealing. Furthermore, in contrast to cell therapy, cell-free exosome therapy is easier to store and produce in large quantities, have fewer risks and pose fewer challenges. Cell-free exosome therapy represents a potentially effective new therapeutic approach is cell-free exosome therapy (Fais et al., 2013; Liu et al., 2018).

Exosomes have been shown in studies to influence tumor growth by establishing an immunosuppressive environment via signal transduction between stromal cells and tumor cells (Zhou Y. et al., 2021). Exosomal microRNA derived from cancerous cells and nearby stromal cells have a tendency to stimulate the development of the metastatic environment (He et al., 2015; Liu et al., 2015; Li et al., 2016; Zhang H. et al., 2017; Plebanek et al., 2017; Yu et al., 2017). Research indicates that exo-miRNA-320a loss in exosomes derived from cancer-associated fibroblasts (CAFs) in HCC can trigger hepatocytes, which are receptor cells, to activate ERK downstream, leading to lung metastasis (Zhang Z. et al., 2017). Likewise, exo-miRNA-1247-3p in exosomes secreted by CAF may facilitate HCC lung metastases (Fang et al., 2018). Additionally, adipocytes can secrete exo-miRNA-23a/b, which can be delivered to cancer cells to stimulate the growth and migration of HCC cells (Liu Y. et al., 2019). According to other research, macrophages can facilitate hepatoma cell invasion by secreting exosomes that contain exo-miRNA-92a-2-5p (Liu et al., 2020). Cancer cells’ exosomes have the ability to influence tumor growth and metastasis. Certain researchers propose that the presence of exo-miRNA-21 and exo-miRNA-10b in the exosomes of HCC, which are generated by an acidic microenvironment, may facilitate the growth and spread of cancerous cells. Thus, they could be employed as HCC therapeutic targets and prognostic molecular markers (Tian et al., 2019). These findings suggest that miRNAs in exosomes can be delivered to target cells within the HCC microenvironment, control the growth of lung cancer cells, and create an environment that is conducive to tumor development and cancer metastasis.

## 2 Roles of microRNA in the liver

### 2.1 MiRNAs in liver metabolic processes

**FIGURE 2:**
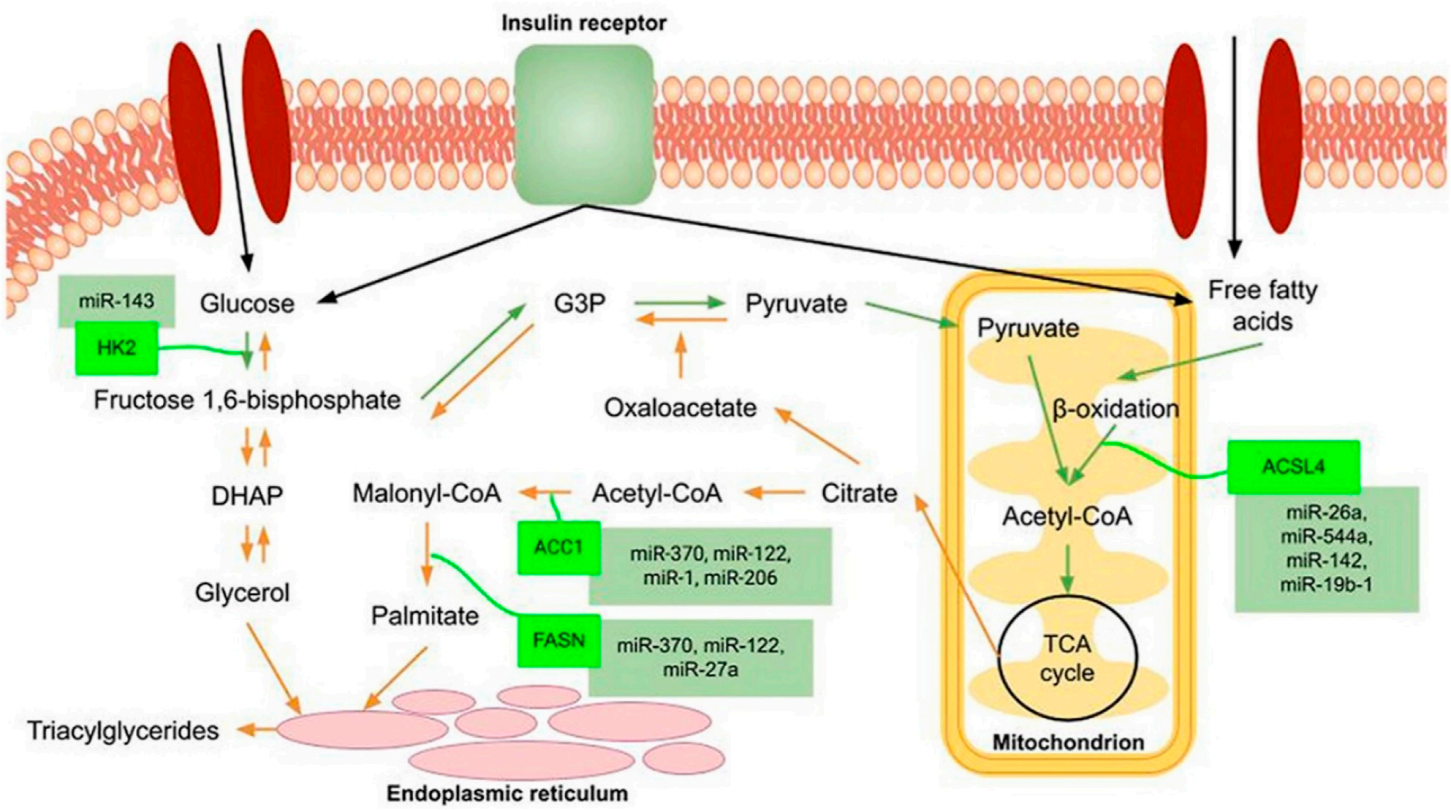
Schematic sketch of signaling pathways linked to carbohydrate and lipid metabolism. The orange arrows depict the part of the pathway that builds glucose and plasma triacylglycerol from the byproducts of the tricarboxylic acid cycle (TCA) and the glycolytic cycle. Some of the representative miRNAs that play vital roles in the metabolism of carbohydrates and lipids are highlighted in green color. Adapted from Paul et al., 2021 with copyright permission, Elsevier.

The liver is a crucial organ in maintaining metabolic homeostasis, playing a central role in both glucose and lipid metabolism. It receives glucose from dietary carbohydrates and releases it from glycogen stores or through gluconeogenesis, ensuring a steady supply to fuel essential tissues like the brain and muscles. Additionally, the liver efficiently processes lipids, absorbing them from the gut, packaging them into lipoproteins for transport, and regulating cholesterol levels to prevent both deficiencies and overabundance (Feingold, 2024). Understanding these intricate metabolic processes in the liver is essential for comprehending metabolic disorders like obesity and diabetes (Adeva-Andany et al., 2016; Trefts et al., 2017). Many miRNAs have emerged and still do as essential regulators in every part of lipid biology. Despite their unfavorable classification as gene expression regulators, loss-of-function investigations in both animal and cell models unequivocally demonstrate the crucial functions that miRNAs play in metabolism, illness, and cellular and animal phenotypes (Figure 2) (Sedgeman et al., 2019; Paul et al., 2021).

According to a recent study by Kaur et al., the increase of gluconeogenesis caused by miRNA-22-3p and its target protein, Tcf7, is critical for the development of diabetes. The results of this investigation confirm miRNA-22 as a novel metabolic regulator and show that it targets Tcf7 to increase the expression of gluconeogenic genes in the liver. These results offer important new information for developing therapeutic approaches that effectively manage diabetes (Kaur et al., 2015). miRNA-206 was shown to be inhibited by fat buildup in the livers of obese mice and human hepatocytes during a study examining the protective effects of miRNA-206 against hepatosteatosis and hyperglycemia. Mice with obesity showed considerable improvements in hepatosteatosis and hyperglycemia after receiving miRNA-206 injections into their livers. Mechanistically, the degradation of PTPN1 (protein tyrosine phosphatase, non-receptor type 1) was caused by miRNA-206s interaction with its 3′untranslated region. Tyrosine kinases and PTPN1 are two different types of enzymes that cooperate to control signaling pathways. One possible therapeutic strategy could be to inhibit PTP1B, SH2, DEP1, and other PTP family members, as they have been connected to a higher risk of developing several human disorders (Verma and Sharma, 2018). miRNA-206 inhibited hepatic lipogenesis by blocking Srebp1c transcription and improved insulin signaling by encouraging phosphorylation of the insulin receptor (INSR) via downregulating PTPN1 expression. In both human hepatocytes and the livers of obese mice, miRNA-206s dual regulation of lipogenesis and insulin signaling led to a decrease in the synthesis of lipids and glucose. miRNA-206s inhibitory effects were reversed in the livers upon reintroducing PTPN1, indicating that PTPN1 is involved in mediating the protective effects of miRNA-206 against hyperglycemia and hepatosteatosis (Wu et al., 2017). According to a study by Castaño et al., lean mice can be efficiently made to develop glucose intolerance, adipose tissue inflammation, and hepatic steatosis by administering exosomes laden with synthetic miRNAs that are similar to those present in the blood of obese mice. These results provide credence to the idea that exosomal miRNAs regulate mice’s metabolism of fats and carbohydrates. Furthermore, the research demonstrated that obesity modifies the miRNA profile of exosomes in circulation in mice, resulting in increased expression of miRNA-122, miRNA-192, miRNA-27a-3p, and miRNA-27b-3p. Therefore, the early phases of the development of the metabolic syndrome, which are marked by the advent of glucose intolerance, dyslipidemia, and central obesity in mice, are actively influenced by obesity-associated exosomal miRNAs (Castaño et al., 2018).

MiRNAs are essential for controlling the metabolism of lipids and glucose in the liver, among other metabolic processes. An array of miRNAs, including miRNA-22-3p, miRNA-206, miRNA-122, miRNA-192, miRNA-27a-3p, and miRNA-27b-3p, have been found to be important modulators of hepatic metabolic pathways. These miRNAs have the potential to affect insulin signaling, gluconeogenesis, lipogenesis, exosome-mediated communication, and ultimately the liver’s general metabolic health. It is crucial to comprehend the processes by which miRNAs control liver metabolism in order to create innovative treatment approaches for metabolic diseases such as diabetes and obesity.

### 2.2 Innate adaptive immunity in hepatic inflammation and anti-inflammatory effects

The liver is an essential component of the immune system that works to keep viruses and other substances out of the body while carefully balancing tolerance and immunity. Maintaining this equilibrium is crucial to avoid both over-inflammatory and inadequate infection control. This balance and general tissue health are largely dependent on the dynamic interactions between different immune cells in the liver (Kubes and Jenne, 2018). The incidence of nonalcoholic fatty liver disease (NAFLD) has increased globally in parallel with the growth in diabetes and metabolic syndrome. NAFLD, a range of liver disorders that includes nonalcoholic steatohepatitis (NASH) and nonalcoholic fatty liver (NAFL), can progress in different ways and result in liver cancer and cirrhosis (Friedman et al., 2018). According to recent research, nonalcoholic fatty liver disease (NAFLD) affects other organs and regulatory mechanisms in addition to the liver. It raises the risk of cardiovascular disease, chronic kidney disease, and type 2 diabetes mellitus. Even though cirrhosis, liver failure, and hepatocellular carcinoma can result from the main liver damage in NAFLD, cardiovascular disease accounts for the majority of NAFLD patients’ fatalities (Byrne and Targher, 2015).

It has been shown that miRNA-26a affects cellular development, differentiation, death, and metastasis. He et al. demonstrated that the miRNA-26a, IL-6, IL-17 axis has an immunoregulatory role in the development of NAFLD. Reduced IL-17 expression and slower NAFLD progression are caused by overexpression of miRNA-26a, which is partly mediated by IL-6 inhibition (He et al., 2017). Through positive regulation of the NF-κB-TNFa axis, miRNA-378 plays a critical role in the development of hepatic inflammation and fibrosis. It has come to light as a possible therapeutic target for NASH management. The incidence of nonalcoholic fatty liver disease (NAFLD) has dramatically increased in correlation with the recent rise in obesity. Effective treatment methods for NAFLD are still inadequate, and the underlying mechanisms are largely unknown. These results show that miRNA-378 stimulates the growth of hepatic fibrosis and inflammation, indicating the therapeutic potential of miR-378 inhibitors for the management of nonalcoholic fatty liver disease (Zhang T. et al., 2019).

### 2.3 Fibrosis signaling pathway

Liver fibrosis, which can lead to cirrhosis, liver cancer, and liver failure, is the body’s wound-healing reaction to liver injury. The primary process in liver fibrosis is the activation of hepatic stellate cells (HSCs). Myofibroblasts and cells generated from bone marrow are further significant elements. The molecular and cellular mechanisms underlying liver fibrosis are poorly understood because the liver is a complex organ (Aydın and Akçalı, 2018). A large number of studies have shown that the expression level of miRNAs in the serum and liver tissue of patients with liver fibrosis has significantly changed (Yu et al., 2023). MiRNAs are implicated in the liver fibrosis process by affecting the proliferation, apoptosis, and activation of HSCs, immune cells, and hepatocytes (Tian et al., 2016).

A study states that the parasite trematode Clonorchis sinensis, which inhabits the bile ducts of animals, releases extracellular vesicles (EVs) that can activate M1-like macrophages and cause biliary damage and fibrosis. This is accomplished by delivering a particular miRNA known as Csilet-7a-5p, which targets the NF-kB(Nuclear factor kappa-light-chain-enhancer of activated B cells) signaling pathway that is regulated by Clec7a (C-type lectin domain family seven member A) and Socs1(Suppressor of cytokine signaling 1) (Na et al., 2020). Tumor formation is dependent on the protein SOCS1, which is targeted by Csilet-7a-5p and is essential for cell signaling and protein breakdown (Yan et al., 2021). Furthermore, another study by Chen et al. discovered that by blocking the mitochondrial fusion protein 2 (MFN2), the elevation of exosomal miRNA-500 in macrophages could accelerate liver fibrosis and encourage the growth and activation of hepatic stellate cells (HSCs). Understanding the role of these molecules in parasite-host interactions could lead to new therapeutic approaches for biliary injuries and fibrosis (Chen et al., 2021). MiRNA-103-3p is present in exosomes produced by THP-1 macrophages that have been treated with lipopolysaccharide (LPS). It works by targeting Krüppel-like factor 4 (KLF4), a transcription factor that is involved in cell division, proliferation, and growth, to promote the activation and growth of hepatic stellate cells (HSCs). The advancement of liver fibrosis is significantly influenced by this interaction between HSCs and macrophages. Exosomes enriched with miRNAR-223 are released more readily in individuals with NAFLD when myeloid cells expressing IL-6 signaling are activated. By transferring antifibrotic miRNA-223 to hepatocytes, these exosomes prevent liver fibrosis and decrease the expression of profibrotic transcriptional activators with PDZ-binding motifs (TAZ) in hepatocytes (Ghaleb and Yang, 2017; Hou et al., 2021). During chronic liver damage, hepatocyte’s miRNA-221-3p activity can be inhibited to facilitate the quick removal of accumulated extracellular matrix and hasten liver healing. Liver fibrosis, a major cause of death from liver illnesses, can be lessened by lowering the levels of miRNA-221-3p in hepatocytes. For liver fibrosis, targeting miRNA-221-3p may be a useful therapeutic approach. Furthermore, hepatocytes exhibiting reduced expression of miRNA-221-3p also exhibit elevated levels of GNAI2 (G protein subunit alpha i2).

A protein that prevents the release of C-C motif chemokine ligand 2 (CCL2). This decrease in hepatic stellate cell (HSC) activation and reduction in liver fibrosis demonstrate the potential therapeutic utility of miRNA-221-3p in liver disorders and its capacity to hasten the clearance of fibrosis (Tsay et al., 2019; PubChem, 2022). The levels of a-SMA and Col1a1, two indicators of liver fibrosis, can be significantly lowered by raising the expression of miRNA-148a-5p in activated LX-2 cells, which are cells that create liver scars. Notch2, a gene implicated in the onset of liver fibrosis, is the target of miRNA-148a-5p. The mechanism by which mesenchymal stem cells (MSCs) provide therapeutic benefits in the treatment of liver fibrosis may be attributed to their ability to enhance the production of miRNA-148a-5p by inhibiting the Notch signaling pathway. The potential of miRNA-148a-5p as a biomarker to track the development of liver fibrosis seems encouraging (Zhou et al., 2022). Increased expression of miRNA-30a can prevent liver scarring by directly inhibiting autophagy, a mechanism that breaks down cellular components, as its levels are low in liver fibrosis. An important modulator of this connection is Beclin1, a protein implicated in both autophagy and apoptosis (Prerna and Dubey, 2022). Consequently, miRNA-30a may provide a novel therapeutic target in the management of liver fibrosis. Because of its anti-fibrotic characteristics, miRNA-30a may be able to treat liver fibrosis by inhibiting the activation of hepatic stellate cells (HSCs), the primary cells in the liver that produce scars. This results in less collagen being produced and more scar tissue breaking down. The research findings indicate that miRNA-30a has an anti-fibrotic effect on HSCs by directly inhibiting Beclin1, which in turn inactivates the Beclin1 signaling pathway and suppresses autophagy in HSCs (Chen J. et al., 2017). It was discovered that three mice models of hepatic fibrosis and activated HSCs treated with TGF-ß1 (Transforming growth factor beta 1) had lower levels of miRNA-488-5p. MiRNA-488-5p was found to decrease HSC multiplication and the expression of fibrosis-related markers *in vitro* tests. Mechanistically, it was found that TET3 mRNA’s 3′UTR is directly bound by miRNA-488-5p, which lowers TET3 (tet methylcytosine dioxygenase 3) protein expression. Consequently, this led to the inhibition of the TGF-ß/Smad2/3 signaling cascade. By suppressing TET3 expression, overexpression of miRNA-488-5p decreased extracellular matrix deposition and ameliorated liver fibrosis in mice (Qiu et al., 2023). The expression of miRNA-150-5p in liver tissue increases with the progression of hepatic fibrosis and decreases with its reversal. Hepatocytes going through apoptosis have an upregulation of this miRNA, whilst proliferating hepatic stellate cells (HSCs) have a downregulation of it. Overexpression of miRNA-150-5p causes hepatocytes to become more susceptible to apoptosis and encourages apoptosis in HSCs. It is interesting to note that HSCs have a stronger effect of miRNA-150-5p on transcriptome stability than do hepatocytes. MiRNA-150-5p is thought to trigger interferon signaling pathways, which could aid in HSC apoptosis. Overall, during liver fibrosis, miRNA-150-5p shows differing regulation and function in hepatocytes and HSCs (Chen et al., 2020). MiRNAs play a crucial role in the development and progression of liver fibrosis. Various miRNAs, including miRNA-103-3p, miRNA-221-3p, miRNA-148a-5p, miRNA-30a, miRNA-488-5p, and miRNA-150-5p, have been shown to regulate the activation, proliferation, and apoptosis of hepatic stellate cells (HSCs), hepatocytes, and immune cells, ultimately influencing the fibrotic process. These findings highlight the potential of miRNAs as novel therapeutic targets for the treatment and management of liver fibrosis.

### 2.4 Exosomal-miRNAs in cellular processes

**FIGURE 3:**
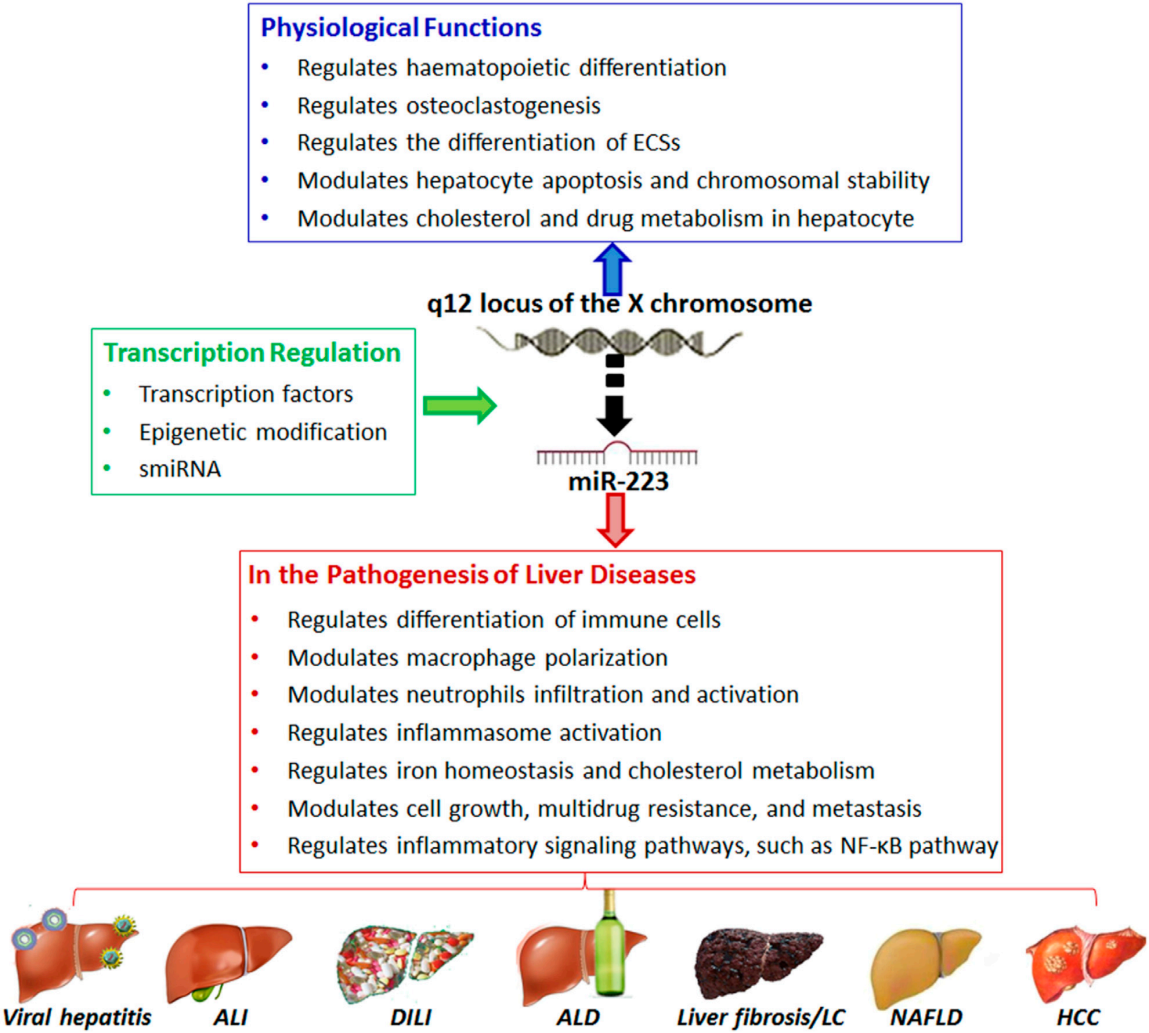
Overview of the role of microRNA (e.g., MiR-223) in normal liver physiology and pathobiology. Adapted from Ye et al., 2018, with copyright permission under the terms of the CC-BY-NC-ND 4.0 license.

MicroRNAs (miRNA) are small, endogenous RNAs that post-translationally regulate gene expression. These RNAs play a pivotal role in the regulation of gene expression and have been increasingly recognized for their involvement in various cellular processes, particularly in the context of HCC (Figure 3) (Ye et al., 2018). In HCC, the aberrant expression of specific miRNAs has been linked to the development and progression of this malignancy. The complexity of their role in HCC becomes evident when considering their interaction with other non-coding RNAs, such as circular RNAs (circRNAs), in the regulation of key molecular pathways (Li et al., 2022).

The circRNAs may play a direct role in miRNA pathways in HCC progression. While circRNAs are generally found to be highly stable and conservative, they can also play multiple functions in disease development, including cancers. Li et al. have shown that the increased expression of circMRPS35, a non-coding circular RNA, directly promotes malignant processes through the inhibition of miRNA-148, therefore inhibiting the miRNA-148a-STX3(Syntaxin 3)-PTEN(Phosphatase and tensin homolog) axis (Li et al., 2022). As miRNA-148 is inhibited, PTEN is consistently ubiquitinated, leading to a decreased expression of pure PTEN, resulting in a promotion of malignant progression. Furthermore, chemotherapy induces the translation of circMRPS35, amplifying the malignant progression while simultaneously developing chemotherapeutic resistance. In another paper by Jiehan et al., miRNA-130b-3p expression was shown to be significantly increased in HCC and downregulated its expression by directly targeting (Homeobox protein Hox-A5), which further activated the PI3K/AKT/mTORpathway, thereby stimulating HCC cells to induce capillary tube formation, endothelial cell migration, and proliferation (Li et al., 2023).

## 3 Diagnostic and therapeutic application of exosomal microRNAs

**FIGURE 4:**
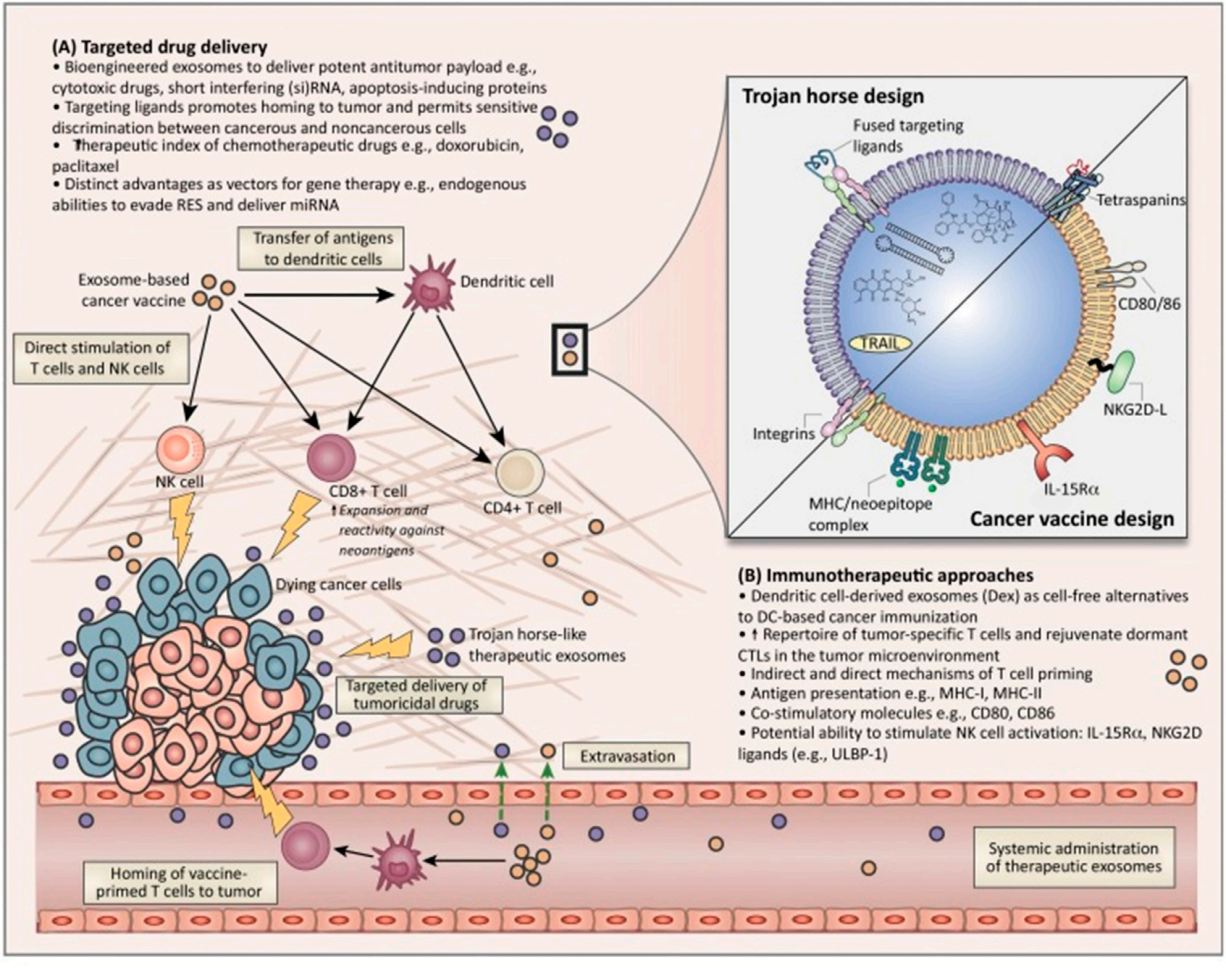
Overview of the potential therapeutic application of exosomes for cancer diagnostics and treatment. Adapted from Syn et al., 2017, with copyright permission, Elsevier. **(A)** Targeted drug delivery approches. **(B)** Immunotheraputic apprachs.

The current research on miRNAs reveals their significant impact on various cellular processes in the pathogenesis and progression of HCC. MiRNAs are found to interact with other non-coding RNAs, such as circular RNAs (circRNAs), influencing pathways related to gene regulation, autophagy, and cellular signaling in HCC. These interactions play a critical role in the development, progression, and therapeutic response of HCC. These insights highlight the potential of miRNAs as biomarkers for early detection, prognostic indicators, and therapeutic targets in HCC (Shen et al., 2016). Understanding the complex roles of miRNAs in HCC opens new avenues for innovative treatment strategies and improved patient outcomes. The ongoing research in this field is crucial for unraveling the intricate molecular mechanisms of HCC and developing more effective, targeted therapies (Figure 4) (Syn et al., 2017; Li et al., 2023).

The use of microRNA as biomarkers for the effectiveness of different HCC treatments is starting to emerge with multiple studies showing promising results. One such study found a difference in expression of nine different miRNAs, such as miRNA-30A, miRNA-122, miRNA-125B, miRNA-200A, and miRNA-374B levels being increased and miRNA-15B, miRNA-107, and miRNA-320B levels decreased, and the complete absence of miRNA645, correlating them to an increased survival benefit with regorafenib and increased overall survival in patients having the Hoshida S3 subtype of the tumor (Teufel et al., 2019). An alteration in the expression of miRNAs has also been determined with the use of Sorafenib, with a comparison of HepG2 cells and primary hepatocytes revealing a differential expression of miRNAs, with nine miRNAs downregulated and 24 miRNAs upregulated in HepG2 cells. These miRNAs are known to target genes involved in cancer-related processes (de la Cruz-Ojeda et al., 2022). Furthermore, the analysis of

Circulating microRNAs revealed that miRNA-200c-3p in patients being treated with sorafenib were predictive of improved survival, whereas increased levels of miRNA-222-5p and miRNA-512-3p after 1 month of treatment were indicative of poorer survival outcomes (de la Cruz-Ojeda et al., 2022).

Another aspect of miRNA could be its use for the overall prognosis of HCC, a research study showed that establishing low levels of miRNA-320d in serum exosomes was associated with more advanced tumor stages, lymph node spread, and poorly differentiated tumors (Li et al., 2020). Patients with lower levels of miRNA-320d in their serum exosomes had shorter overall and disease-free survival. Low levels of miRNA-320d in serum exosomes were independently associated with a worse prognosis for HCC. In addition, overexpression of miRNA-320d in HCC cells inhibited their proliferation and invasion, and BMI1 was shown to be a direct target of miRNA-320d (Li et al., 2020). Another study highlighted that low serum miRNA-122 has a strong association with poor progression-free survival and overall survival, although predicting overall survival is not possible with serum miRNA-122 levels (Zhang Y. et al., 2019). However, there has been strong evidence arguing the correlation between CHST4, SLC22A8, STC2 (Carbohydrate sulfotransferase 4, Solute carrier family 22 member 8, Stanniocalcin-2), hsa-miRNA-326, and hsa-miRNA-21 with a strong potential for predicting prognosis in HCC patients specifically with sa-miRNA-326 andhsa-miRNA-21-5p have been found to have associations with multiple cancer-related pathways (Hu et al., 2021).

The likelihood of developing HCC increases exponentially in the event of an HCV infection, which becomes of great importance in prevention and control. MiRNA expression is altered in the event of an HCV infection, such as miRNA-135 having a “proviral” effect due to its ability to increase HCV RNA replication in hepatocytes (Badami et al., 2022). Furthermore, miRNA-135a has been shown to suppress the expression of CXCL2, MyD88, and IRPK2 (Chemokine (C-X-C motif) ligand 2, Myeloid differentiation primary response 88 and Receptor-interacting serine/threonine-protein kinase 2) which are host restriction factors that are essential components of the antiviral immune response (Sodroski et al., 2019). Another example is miRNA-146a-5p with its dual function of downregulation of inflammatory signaling and inhibiting the hepatocyte immune response (Badami et al., 2022)**.**

## 4 Exosomal RNA biomarker isolation strategies

### 4.1 Isolation and detection of exosomes

Most of the miRNAs are known to bind with free protein present in the body fluids. Detection of miRNAs without isolation of EV is complicated. Therefore, it is necessary to isolate the Evs from the body fluids and use different methods to quantify the EV-derived miRNAs (Hu et al., 2018; Yang et al., 2018). Isolation of exosomes is very important for the detection of exosomal-miRNAs there are many methods available for the isolation of exosomes. However, the following methods such as ultracentrifugation (UC), size exclusion chromatography (SEC), density gradient centrifugation (DGC), immunoaffinity, and co-precipitation are used often to isolate the exosomes (Xu et al., 2022). Recently aptamers specific to the exosome membrane proteins have been used as targets to capture the exosomes (Chinnappan et al., 2023a; Chinnappan et al., 2023b).

### 4.2 Centrifugation methods

The separation of exosomes by UC is based on the physical and chemical properties of exosomes. This is a gold standard classical method for exosome isolation. Differential ultracentrifugation method is used for the separation of exosomes from other biological components. Despite its wide use, it has many limitations such as high-cost instruments, aggregation of exosomes and sticking with other components, high-speed centrifugation leads to morphological changes in the exosomes. Exosome isolation from density gradient centrifugation has high resolution and high purity. UC and DGC methods can not be used for the isolation of exosomes from large volumes of biological fluids and to detect the exosomal miRNAs.

### 4.3 Size exclusion chromatography(SEC)

The isolation of exosomes from the SEC method is based on the particle size. The exosome passes through the pore size of the polymer beads loaded in the chromatographic column. The particles with a small radius will move rapidly and the particles with a large size cannot enter into the polymer pore. Exosome isolation from SEC is pure without soluble components and viruses, and proteins. Therefore this method is more suitable for clinical applications and basic research.

### 4.4 Ultrafiltration

This is a simple and efficient method for the isolation of exosomes. It will not alter the morphology or the biological behavior of the particles. In this method, a membrane with a specific pore size is used to collect exosomes. This method will be useful for the isolation of exosomes from large volumes of biological samples. This method separates the exosome only by particle size and therefore, it cannot remove all the impurities and it is not specific.

### 4.5 Co-precipitation

This method of polymer co-precipitating agent reduces the solubility of exosomes significantly, and as a result, it precipitates easily. This method is very simple and rapid. The isolation efficiency is 2.5-fold higher than the ultracentrifugation technique. This method cannot be used for large-scale applications it also co-precipitates with organelle-related proteins. The addition of precipitating reagents are contaminants along with exosomes, therefore the isolated exosomes limit the further application of isolated exosomes**.**

### 4.6 Immunoaffinity enrichment

This is an efficient method for the isolation of specific exosomes. The specific antibodies of exosome-specific biomarkers, such as CD6, CD63, CD81, EGFR, and EpCAM are immobilized with magnetic beads, chips, and ELISA plates. The immunoaffinity capture methods can specifically bind to the exosome component resulting in isolation of specific and pure exosomes. The separation of exosomes from the solid support is challenging due to the strong interaction between the antibody and antigen. In addition to antibodies, exosome component-specific aptamers are also used for the isolation of exosomes and further analysis (Chinnappan et al., 2023a).

### 4.7 Field flow fractionation (FFF)

Unlike size exclusion chromatography, the FFF method works in a single phase. The sample flows in the FFF channel in a parabolic way. The vicious particles stay in the center and the particles with less viscosity will move closer to the channel wall (Zhang and Lyden, 2019). This method is ideal for the separation of different particle sizes. The sample preparation for this method is tedious, it is time-consuming and limits the wide application.

### 4.8 Acoustic-based isolation method

The Acoustic-based microfluidics separation of exosomes is highly precise. The ultrasonic wave is used for the separation of the particles. Under sound pressure, the particles are separated based on their characteristic physical properties, such as size and density. This is a rapid, label-free, contact-free method of exosome isolation (Hassanpour Tamrin et al., 2021).

### 4.9 Absorbent polymer-based method

This method is based on the high water-absorbing ability of the hydrogels. In the presence of hydrogel, the small molecules will be absorbed into the pore sizes of the hydrogel, and the exosomes and large-size particles will be excluded for the concentration and purification. Yang et al. have successfully enriched the exosomes by this method from culture media and urine (Yang et al., 2021). The interaction between the hydrophobic surfaces and the microbes in the urine sample can be utilized for concentrating the microorganisms. Tuning the hydrophobicity surfaces was acting as a sensing platform for the detection of nucleic acid and other metabolites (Sudarsan et al., 2023; Uttam et al., 2024). A similar methodology was used for the isolation of exosomes. For example, Hydrophobic interaction chromatography technology was used for the isolation of exosomes from urine and plasma samples using a polyester, capillary-channeled polymer fiber phase (Wang L. et al., 2019; Huang et al., 2019).

## 5 Exosomal microRNA detection methods

There are several methods developed for the quantitative and qualitative detection of exosome-derived miRNAs. The reverse transcription polymerase chain reaction (qRT-PCR) method is the gold standard for the quantitative detection of exosomal-miRNAs. In addition, there are many other methods such as surface-enhanced Raman scatting (SERS), microarray, molecular beacon fluorescence assay, isothermal amplification, and next-generation sequencing methods that have been developed. Most of the methods use probe molecules or complementary primers for the detection of miRNAs.

### 5.1 Quantitative reverse transcription polymerase chain reaction (qRT-PCR)

Exosomal miRNAs quantification by qRT-PCR consists of two steps. In the first step, the complementary DNA (cDNA) of the target, miRNAs will be produced by reverse transcription processes. In the second step, the cDNA will be used as a template for the real-time- PCR amplification which is monitored by changes in the fluorescence of the probe dye with time (Chinnappan et al., 2019). There is a standard reference for the exosomes that is used for the quantification of exosomal-miRNAs because there is no stable expression of miRNAs in the exosomes that can be used as standards. Magnetic nanoparticle-based Portable nucleic acid detection (PNAD) has been designed by the integration of sample processing and PCR amplification in a single device. This device can work in three different modes such as high-precision heating rapid thermal cycle control and rate-adjustable constant heating/cooling control for nucleic acid extraction, PCR, and melting curves respectively (Fang et al., 2021). Droplet digital PCR(ddPCR) is an advanced nucleic acid amplification technology, that is highly precise and accurate in the quantification of nucleic acid. The outstanding performance of ddPCR was noticed for the quantification of miRNAs from serum samples for the diagnosis of cancer (Hindson et al., 2013). Wang et al. demonstrated that the quantitative detection of exosomal-miRNAs from urine samples by ddPCR exhibits an excellent sensitivity compared to the conventional qPCR. It could detect miNA-29A as low as 50 copes/µL (Wang C. et al., 2019). Exosome-derived miRNAs from endometrial cancer (EC) patient’s plasma samples have been quantified by PCR method. It has been found that the miRNA-15a-5p, miRNA-106b-5p, and miRNA-107 were upregulated compared to healthy individuals (Zhou L. et al., 2021).

### 5.2 Insitu detection of miRNAs by molecular beacons

Fluorescence assay is used for the *in-situ* detection of target nucleic acids specifically. Several types of molecular beacons were used for the sensitive detection of miRNAs and other RNA targets (Raja et al., 2006; Chinnappan et al., 2013; Chinnappan et al., 2019). The molecular beacon consists of a stem-loop DNA with a fluorophore and a quencher attached at the 5′and 3′ends of the stem, which can bind to the target RNA specifically and regenerate the fluorescence. The increase in the fluorescence intensity is directly proportional to the quantity of miRNAs present in the sample. Lee et al. have designed two different molecular beacons for the simultaneous detection of two miRNAs (such as miRNA-375 and miRNA- 574-3P) specific to prostate cancer. The urine samples were used directly for the quantification of miRNAs without any sample processing steps (Lee et al., 2018). Lee et al. have demonstrated the *in situ* single-step detection of exosomal miRNA--21 specific for breast cancer using the molecular beacon probe from the patient serum sample (Lee et al., 2015). Many other exosomal miRNAs were detected using the target-specific molecular beacon (Xu et al., 2022). There is more possibility of false positive results due to the autofluorescence, low abundance of the target miRNAs leads to more noise and light scattering due to the inhomogeneity of the samples.

### 5.3 Microarray

The microarray assay is based on the hybridization of the predesigned probe to the target sequences. The total RNA extracted from the samples will be labeled with a fluorescent probe and hybridized with the complementary DNA which is immobilized on the glass slide. The signal intensity after hybridization is correlated with the quantity of miRNAs in the sample. The fluorescence emission from different kinds of miRNA hybridizes with the respective probes at different positions can be detected. From the signal intensity and the position, the nature of miRNAs and their quantity can be determined. Exosome-derived miRNAs from type-1 autoimmune pancreatitis (AIP) samples, chronic pancreatitis (CP), and healthy adults (HA) were analyzed. The over-expression of miR-21-5p was observed compared to healthy adults (Nakamaru et al., 2020). Two hundred and ten different exosome-derived miR expression patterns were identified using TaqMan open-array technology from peritoneal lavage fluid of patients suffering from colorectal cancer (CRC) (Roman-Canal et al., 2019). Different expression levels of Alzheimer’s disease (AD) specific miRNAs have been studied using 5XFAD mouse model. The microarray analysis showed that 48 miRNAs expressed differently, of which six miRNAs played play important role in gene targets and signaling pathways of AD (Song et al., 2021). Despite the multiplex analysis, microarray technology has certain limitations such as low sensitivity, expensive, and narrow range of detection.

### 5.4 Next-generation sequencing (NGS)

NGS is an advanced technology for high-throughput sequencing in the transcriptome. It can be used for sequencing DNA or RNA base pairs. The total RNA from the sample is to be purified and the universal adaptor has to be connected to both 5′and 3′ends of RNA strands followed by reverse transcription, PCR amplification, and sequencing (Miller et al., 2022). NGS has more advantages over microarrays such as high sensitivity and accuracy and many unknown miRNAs can be detected. NGS is often used for the detection of miRNAs for specific diseases. Overexpression of exosome-derived miRNA-10a-5P and miRNA-29b-3P from a prostate cancer patient’s plasma samples by NGS technology (Worst et al., 2019). This methodology can detect new sequences, however, it is not apt for standard detection due to high cost and complex data analysis.

### 5.5 Isothermal amplification technique

This technique is one of the easy and simple methods for the detection of miRNAs. This method allows the amplification of nucleic acids at a constant temperature without the aid of a thermocycler. This is suitable for the detection of short sequences like miRNAs (Gines et al., 2020). There are two kinds of isothermal amplifications such as with enzyme and enzyme-free. The enzymatic amplification includes loop-mediated isothermal amplification (LAMP), nuclear acid sequence-based amplification (NASBA), rolling circle amplification (RCA), and exponential amplification reaction (EXPAR). RCA technology was used for the sensitive detection of miRNA-21,miRNA-122, and miRNA-155 from exosomes simultaneously (Wang et al., 2020). Catalytic hairpin assembly (CHA) and hybrid chain reaction (HCR) are enzyme-free methods. Electrochemical detection of miRNA-122 was demonstrated using the HCR amplification method (Guo et al., 2020).

### 5.6 Clinical application of exosome-derived miRNAs

The exosome-mediated intercellular transmission of miRNAs exhibits a new model in the clinical research area. The short non-coding RNA can be transferred from 1 cell to another cell through exosomes and create an RNA-induced silencing complex (RISC) which could cause the degradation of target mRNA or prevent the protein translation. Therefore, exosomal-miRNAs play an important role in the gene regulation pathways in the recipient cells (Ghafouri-Fard et al., 2023). The exosome-derived miRNAs and their influence on various disorders, including pulmonary, neurological, and cardiovascular disorders, gastrointestinal disorders, and cancers. Serum Exosome derived-MiR-638 was identified as a significant and independent prognostic biomarker for HCC. The overexpression of exosomal miRNA-638 was associated with the reoccurrence of the tumor. The cancer cell-secreted miRNAs promote vascular permeability by downregulation of endothelial expression of VE-cadherin and ZO-1 (Yokota et al., 2021). Mesenchymal stem cells secreted exosomal-miRNA-15a hinder the progression of HCC by down-regulating the Sal-like protein 4 (SALLA4) levels (Ma et al., 2021). The serum exosomal miRNA-720 is identified as an excellent biomarker for the detection of HCC, which gives more accurate results compared to AFP or PIVKA-II (protein induced by vitamin K absence). The exosomal miRNA-720 is not influenced by the aminotransferase levels (Jang et al., 2022). There are several other miRNAs are utilized as potential biomarkers in the clinical application for the diagnosis of HCC. The major limitations are the following, there is no standardized method for the detection of exosome miRNA. Very limited numbers of clinical samples were used for the studies and the experimental settings and the detection methodologies vary from lab to lab. There is no standard optimized method for the isolation of exosomes. Most of the studies were conducted using serum and blood samples, however, most of the biological fluids contain exosomes. Therefore, more studies have to be done using other biological fluids. Another big challenge is the production of a large quantity of exosomes for clinical trials. 3D scaffold or microfluidic can be used for the alrge production, however, the purity of the isolated exosomes are another upto the clincal levels using this methods, other kinds to Evs and the miomolecules of exosome sizes will be contaminated. Various types of exosomes and their complexicity make the miRNA based HCC diagnosis more challenging.

## 6 Conclusions and future prospectives

MicroRNAs have been attracted recently due to their potential as a biomarker for the detection of cancer and other diseases, they are also used as predictive prognosis. Despite much research has been done on miRNAs, and specific features of miRNAs roles; still it is under investigation including sample preparation methods, analysis and selection of the control. There are several variables to consider when studying miRNAs and their role as biomarkers and mediators of disease. The sex, age, and body mass index of the patient may result in significant variation in miRNA levels. The samples used as health control are often questionable. The age and sex-matched control sample used for the study may not have the diseases of interest, yet, it is not clear that the miRNAs are associated with age and sex. Moreover, some other disease factors may be a match between the control sample and the disease samples (Takizawa et al., 2022). In most cases, these issues have not been highlighted, there are very limited reports that have considered these issues. A study by Ameling et al.demonstrated that expression levels of 179 miRNAs from 372 healthy volunteers were selected from a previous population-based cohort study. There are 12 and 19 miRNAs that were significantly associated with age and BMI after adjusting the blood cell parameters. Out of 35 associated miRNAs, it was reduced to 7 after adjustment with age, BMI, and blood cell parameters (Ameling et al., 2015). Additionally, there is a great lack of standardized protocols for the collection and processing of samples for miRNA studies (Takizawa et al., 2022). Studies may use either plasma or serum, which could introduce variations across studies. For example, Binderup et al. demonstrated significant differences between miRNA levels in re-centrifuged biobank plasma compared to platelet-poor plasma (Binderup et al., 2016). Finally, miRNA extraction methods, such as next-generation sequencing and reverse transcriptase quantitative polymerase chain reaction, result in method-dependent variation (Kloten et al., 2019). Hence, it is important to take these variables into consideration when analyzing the role of miRNAs in HCC and interpreting potentially conflicting data in the field. Future research should focus on overcoming these challenges through developing efficient isolation techniques, standardizing detection methods, and conducting extensive clinical trials.
